# Cholecystokinin-2 Receptor Targeting with Novel C-terminally Stabilized HYNIC-Minigastrin Analogs Radiolabeled with Technetium-99m

**DOI:** 10.3390/ph12010013

**Published:** 2019-01-15

**Authors:** Maximilian Klingler, Christine Rangger, Dominik Summer, Piriya Kaeopookum, Clemens Decristoforo, Elisabeth von Guggenberg

**Affiliations:** Department of Nuclear Medicine, Medical University of Innsbruck, Anichstrasse 35, A-6020 Innsbruck, Austria; maximilian.klingler@i-med.ac.at (M.K.); christine.rangger@i-med.ac.at (C.R.); dominik.summer@i-med.ac.at (D.S.); piriya.kaeopookum@student.i-med.ac.at (P.K.); clemens.decristoforo@i-med.ac.at (C.D.)

**Keywords:** cholecystokinin-2 receptor, minigastrin, molecular imaging, radiometals, technetium-99m, hydrazinonicotinic acid (HYNIC)

## Abstract

The high overexpression of cholecystokinin-2 receptors (CCK2R) in tumors, such as medullary thyroid carcinoma, allows for highly specific diagnostic and therapeutic targeting with radiolabeled peptide probes derived from natural ligands for the receptor. Based on the ideal imaging characteristics, high availability and low cost of technetium-99m (^99m^Tc)-labeled radiopharmaceuticals we have developed two hydrazinonicotinic acid (HYNIC) conjugated minigastrin analogs allowing labeling at high specific activity. The CCK2R targeting peptide conjugates show specific amino acid substitutions in the C-terminal receptor-specific sequence with the aim to increase stability and tumor targeting. The CCK2R affinity and the cell uptake of the new radioligands were analyzed using A431 human epidermoid carcinoma cells stably transfected with human CCK2R and mock transfected cells. Metabolic studies in BALB/c mice revealed a high resistance against enzymatic degradation for both radioligands. Biodistribution studies in tumor-xenografted athymic BALB/c nude mice at 1 h and 4 h p.i. showed that the two ^99m^Tc-labeled compounds showed varying uptake in receptor expressing organs, stomach and pancreas (1.3–10.4% IA/g), as well as kidneys, the main route of excretion (7.8–19.9% IA/g). The tumor uptake in A431-CCK2R xenografts was 24.75 ± 4.38% IA/g for [^99m^Tc]Tc-HYNIC-MGS5 and 42.48 ± 6.99% IA/g for [^99m^Tc]Tc-HYNIC-MGS11 at 4 h p.i., whereas the tumor-to-kidney ratio was comparable (2.6–3.3). On demand availability and potential application for radioguided surgery of a ^99m^Tc-labeled minigastrin analog support the further evaluation of these highly promising new compounds.

## 1. Introduction

Regulatory peptides exhibiting high target specificity are suitable lead structures, particularly in the field of oncology, for the development of analogs for radionuclide imaging and therapy [1,2]. Such peptide analogs have the advantage of an easy production via well-established solid phase peptide synthesis (SPPS) allowing the conjugation of a chelator for radiolabeling and the introduction of different modifications into the peptide sequence to optimize pharmacokinetics. Additionally, peptide analogs show low immunogenicity and toxicity and are therefore preferable candidates as new targeting agents [3,4]. A highly promising molecular target to develop radiopeptides for diagnostic imaging and targeted radiotherapy (TRT) of medullary thyroid carcinoma (MTC), small cell lung cancer (SCLC), astrocytoma, stromal ovarian cancer, as well as carcinoids and other tumors of neuroendocrine origin is the cholecystokinin-2 receptor (CCK2R) as an increased level of expression is observed in these malignancies [5,6].

The most promising CCK2R targeting radiopeptides developed so far, are based on the peptide sequence of minigastrin (MG), a naturally occurring ligand for this receptor [7]. MG and its analogs bind to CCK2R with their bioactive C-terminal region (Trp-Met-Asp-Phe-NH_2_). In the last 20 years a variety of MG analogs conjugated to different chelators for nuclear medicine procedures have been reported [7,8,9]. Most of the preclinically [10,11,12,13,14,15,16] and clinically [17,18] investigated MG analogs have been conjugated to the bifunctional chelator 1,4,7,10-tetraazacyclododecane-1,4,7,10-tetraacetic acid (DOTA) allowing stable radiolabeling with trivalent radiometals such as Gallium-68, Indium-111 or Lutetium-177 suitable for positron emission tomography (PET), single photon emission computed tomography (SPECT) and TRT. Due to radioprotection issues and regulatory requirements for the in-house production of radiopharmaceuticals, such as the need of hot cells, automated synthesis modules, aseptic processing and trained personnel, the availability of these radiopeptides seems to be restricted to a limited number of clinics.

A cost-effective and broadly available alternative for CCK2R imaging in hospitals without PET would be a kit-based MG analog suitable for ^99m^Tc-labeling. Such a kit is already available for somatostatin receptor targeting using [^99m^Tc]Tc-EDDA/HYNIC-Tyr^3^-octreotide (Tektrotyd, Polatom, Otwock, Poland). Technetium-99m can be easily eluted from a licensed ^99^Mo/^99m^Tc-generator and is used in the major part of nuclear medicine procedures. Due to its ideal physical properties (half-life of 6 h, monoenergetic gamma photons of 140 keV, low radiation burden) Technetium-99m remains the most attractive radionuclide for SPECT applications as well as for gamma probe detection during radioguided surgery [19,20].

Different attempts have been made to develop MG analogs with high tumor accumulation and a biodistribution profile suitable for gastrin receptor scintigraphy [19,21,22]. However, only two ^99m^Tc-labeled MG analogs have been investigated in clinical studies. [^99m^Tc]Tc-Demogastrin 2 ([^99m^Tc]Tc-N_4_-Gly-dGlu-(Glu)_5_-Ala-Tyr-Gly-Trp-Met-Asp-Phe-NH_2_) contains an open chain tetraamine chelator forming a monocationic complex [23,24]. [^99m^Tc]Tc-EDDA/HYNIC-MG11 ([^99m^Tc]Tc-EDDA/HYNIC-dGlu-Ala-Tyr-Gly-Trp-Met-Asp-Phe-NH_2_) conjugated to the monodentate ligand hydrazinonicotinic acid (HYNIC) needs additional coligands such as tris(hydroxymethyl)-methylglycine (tricine) or ethylenediamine-*N*,*N*’-diacetic acid (EDDA) to complete the coordination sphere [25]. The administration of these two ^99m^Tc-labeled MG analogs to patients was well tolerated showing no to only mild side effects. With [^99m^Tc]Tc-Demogastrin 2 all known lesions in nine MTC patients could be visualized. In a comparative study with [^99m^Tc]Tc-EDDA/HYNIC-MG11 and [^99m^Tc]Tc-EDDA/HYNIC-TOC the potential additional information which can be obtained in MTC patients using gastrin receptor scintigraphy was pointed out. The same MG analogs conjugated to DOTA showed drawbacks related to high kidney uptake or low in vivo stability, requiring further improvement to develop a CCK2R targeting peptide analog with optimal tumor targeting and biodistribution profile [8].

Various research groups have worked on the development of metabolically stable MG analogs. Different strategies such as cyclization, dimerization and substitutions of amino acids mainly in the N-terminal part of the peptide sequence were investigated [8]. However, due to rapid C-terminal enzymatic degradation the need of alternative stabilization strategies was suggested [26]. Recently we could present different amino acid substitutions introduced into the C-terminal receptorspecific sequence of MG analogs improving the stability against enzymatic degradation and the biodistribution profile [15,16]. After an intense preclinical evaluation of different substitutions, we discovered that most promising results in terms of improved in vivo stability and enhanced tumor targeting could be achieved when substituting methionine (Met) with *N*-methyl-norleucine ((*N*-Me)-Nle) and phenylalanine (Phe) with 1-naphtyl-alanine (1-Nal). With the new MG analog DOTA-dGlu-Ala-Tyr-Gly-Trp-(*N*-Me)Nle-Asp-1-Nal-NH_2_ (DOTA-MGS5) radiolabeled with Gallium-68, Indium-111 and Lutetium-177 a very promising targeting profile was achieved [16]. In the present study we have conjugated the clinically well-established HYNIC ligand to MGS5 to develop a ^99m^Tc-labeled MG analog, suitable for SPECT and radioguided surgery. Furthermore the HYNIC-conjugate dGlu-Ala-Tyr-Gly-Trp-(*N*-Me)Nle-Asp-(*N*-Me)1-Nal-NH_2_ (HYNIC-MGS11) with additional N-methylation of the peptide bond between Asp and 1-Nal was synthesized to evaluate if a further stabilizing effect can be achieved. The two ^99m^Tc-labeled MG analogs were characterized in vitro and in vivo, including receptor affinity and cell uptake assays, as well as metabolic and biodistribution studies in tumor-xenografted BALB/c nude mice.

## 2. Results and Discussion

### 2.1. Peptide Synthesis and Radiolabeling 

**Figure 1:**
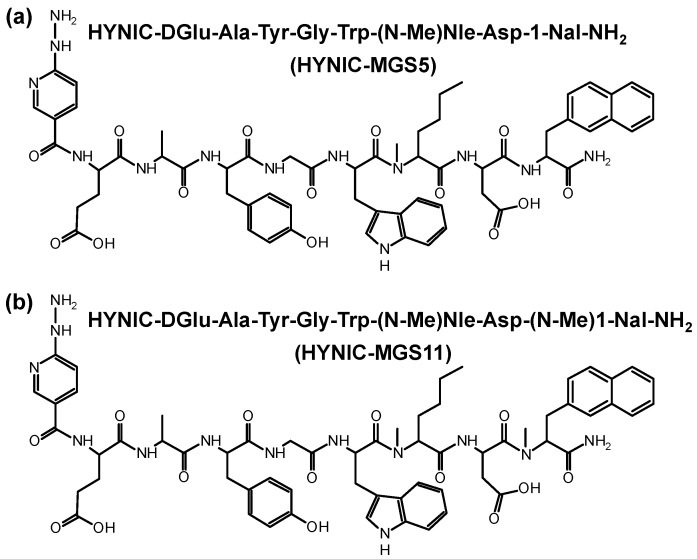
Amino acid sequence and chemical structure of (**a**) HYNIC-MGS5 and (**b**) HYNIC-MGS11.

Following straightforward SPPS HYNIC-MGS5 and HYNIC-MGS11 were synthesized using 30 µmol of resin, 150 µmol of each Fmoc-protected amino acid and 90 µmol of HYNIC. After purification by RP-HPLC and lyophilization the peptide conjugates were obtained in ~10% yield with a chemical purity ≥95% as confirmed by RP-HPLC and MALDI-TOF MS. The amino acid sequences and chemical structures of both MG analogs are presented in Figure 1.

**Figure 2:**
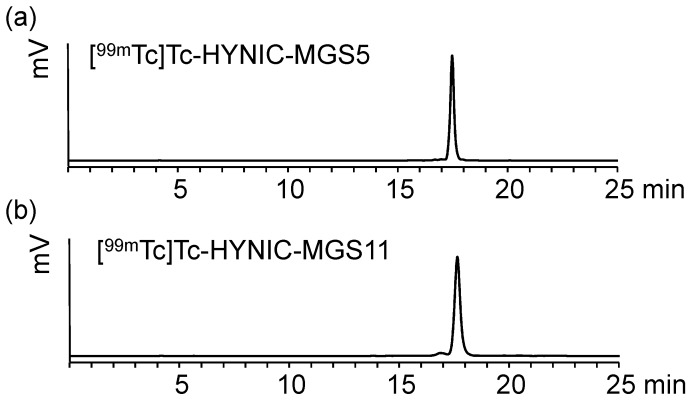
Radiochromatograms of (**a**) [^99m^Tc]Tc-HYNIC-MGS5 and (**b**) [^99m^Tc]Tc-HYNIC-MGS11.

Radiolabeling with technetium-99m using the exchange labeling approach from tricine, used as an intermediate coligand, to EDDA yielded in [^99m^Tc]Tc-HYNIC-MGS5 and [^99m^Tc]Tc-HYNIC-MGS11 at high molar activity of 35–40 GBq/µmol comparable to previously published results [22,27]. The main peak occurring in the radio-HPLC chromatogram after labeling indicates complete conversion of the initial tricine complex into the EDDA complex, as already described previously [28,29]. Minor hydrophilic impurities, related to free pertechnetate and ^99m^Tc-coligands, could be efficiently removed by solid phase extraction (SPE) resulting in labeling with radiochemical purity >95%. Peptide related side products with relative retention of 0.9–1.1 were below 5%. Representative radiochromatograms are displayed in Figure 2.

### 2.2. Characterization in Vitro

**Figure 3:**
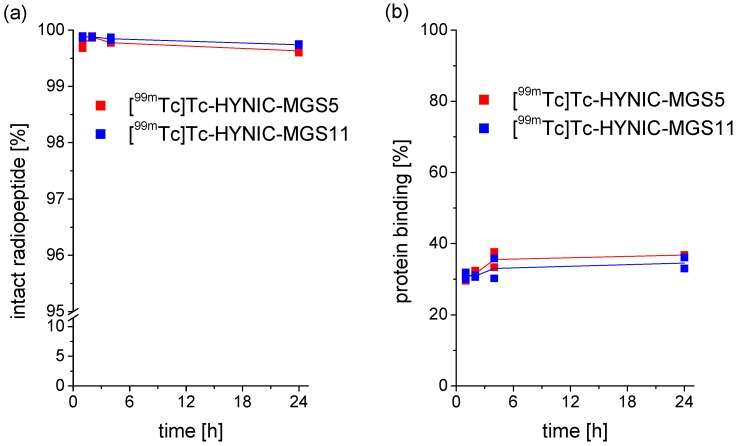
In vitro properties of [^99m^Tc]Tc-HYNIC-MGS5 (red) and [^99m^Tc]Tc-HYNIC-MGS11 (blue): (**a**) stability in human serum (*n* = 2), (**b**) protein binding (*n* = 2).

The stability of the two radiopeptides was analyzed in PBS, confirming a high complex stability obtained by exchange labeling. After 24 h incubation, still a high percentage of intact radiopeptide was present ([^99m^Tc]Tc-HYNIC-MGS5: 95.1%; [^99m^Tc]Tc-HYNIC-MGS11: 94.4%). MG analogs missing the penta-Glu motif are generally known for their low in vitro serum stability regardless of whether they are conjugated to DOTA [26] or HYNIC [30]. This can be changed by introducing site-specific amino acid substitutions into their C-terminal peptide sequence [15,16]. For [^99m^Tc]Tc-HYNIC-MGS5 and [^99m^Tc]Tc-HYNIC-MGS11 showing such substitutions, a high stability with no evidence of enzymatic degradation was found in human serum (>95% intact radiopeptide after 24 h incubation). These results are in accordance with previous results reported for ^111^In-, ^68^Ga- or ^177^Lu-labeled DOTA-MGS5 (96–98% intact radiopeptide) incubated under the same conditions [16]. From the octanol/PBS distribution a log D value of −2.91 ± 0.06 was calculated for [^99m^Tc]Tc-HYNIC-MGS5. The additional methyl group in [^99m^Tc]Tc-HYNIC-MGS11 did not change the hydrophobicity (log D value of −2.84 ± 0.08; *p* = 0.07). Protein binding with values of 36.8 ± 0.1% for [^99m^Tc]Tc-HYNIC-MGS5 and 34.5 ± 2.2% for [^99m^Tc]Tc-HYNIC-MGS11 after 24 h incubation was in the same range. The results of serum stability and protein binding observed over the incubation period of up to 24 h are summarized in Figure 3.

### 2.3. Receptor Binding and Cell Internalization Studies

**Figure 4:**
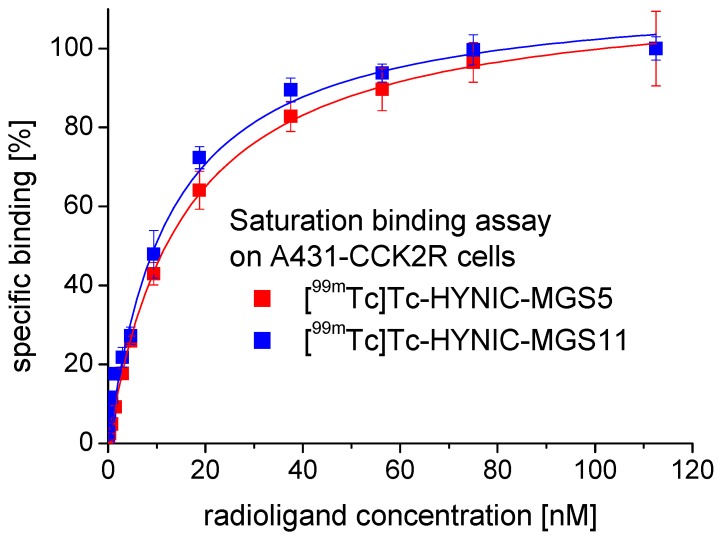
Representative saturation binding curve obtained on A431-CCK2R cells with [^99m^Tc]Tc-HYNIC-MGS5 (red) and [^99m^Tc]Tc-HYNIC-MGS11 (blue).

Saturation binding experiments on A431-CCK2R cells revealed a high affinity to the human CCK2R. The mean values for the dissociation constant (*K*_d_) calculated by fitting the data from two experiments to a one-site binding model (r² ≥ 0.99) were 13.7 ± 1.1 nM for [^99m^Tc]Tc-HYNIC-MGS5 and 14.7 ± 1.2 nM for [^99m^Tc]Tc-HYNIC-MGS11. In Figure 4 a representative saturation binding curve is displayed for both radioligands.

**Figure 5:**
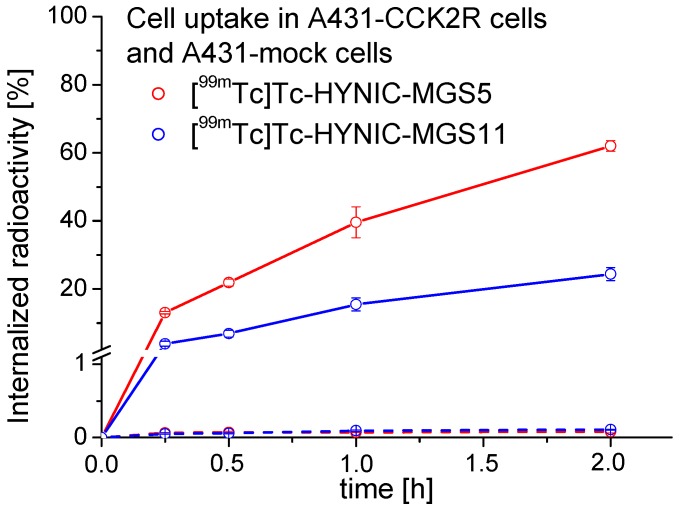
Cell uptake of [^99m^Tc]Tc-HYNIC-MGS5 (red) and [^99m^Tc]Tc-HYNIC-MGS11 (blue) into A431-CCK2R (solid line) and A431-mock (dashed line) cells for up to 2 h incubation.

In the internalization assays performed on A431-CCK2R cells a high receptor mediated cell uptake was observed. With [^99m^Tc]Tc-HYNIC-MGS5 13.1 ± 0.4% of the totally added radioactivity was internalized already after 15 min and this value increased over time reaching 62.0 ± 1.6% after 2 h incubation. Even though [^99m^Tc]Tc-HYNIC-MGS5 and [^99m^Tc]Tc-HYNIC-MGS11 showed very similar *K*_d_ values, the cell uptake of [^99m^Tc]Tc-HYNIC-MGS11 was distinctly lower. After 15 min 4.0 ± 0.7% of the radioactivity was internalized and this value increased to 24.4 ± 1.9% after 2 h incubation. These unexpected differences between the two MG analogs indicate a possible different binding mode to human CCK2R of MGS5. Also for DOTA-conjugated MGS5 labeled with different radiometals a similarly enhanced cell uptake was found [16]. The results of the internalization experiments are displayed in Figure 5. 

In A431-mock cells lacking CCK2R expression a low and comparable non-specific uptake of radioactivity (≤0.1%) was observed for both radiopeptides at each time point. The receptor specificity of the uptake in A431-CCK2R cells could be confirmed by additional blocking studies with 1 µM pentagastrin showing a clear blockage of the CCK2R mediated uptake to values ≤1.0% after 2 h incubation for both radiopeptides (data not shown).

### 2.4. In Vivo Stability in BALB/c Mice

**Figure 6:**
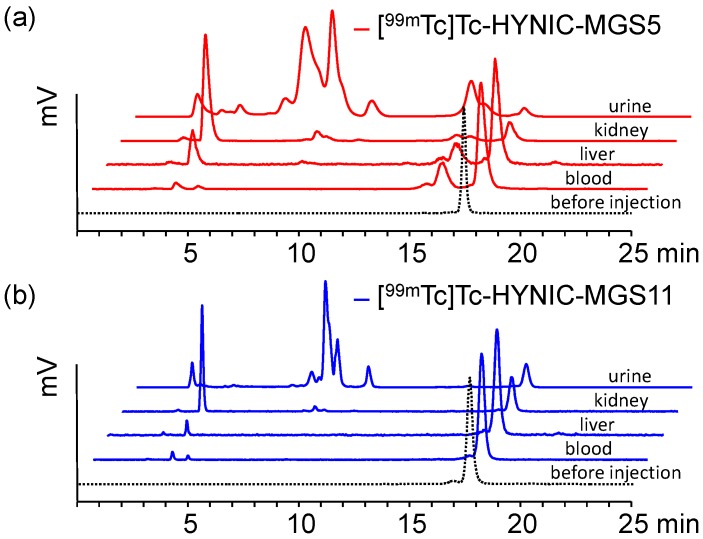
Radiochromatograms for blood, liver, kidney and urine from metabolite studies in BALB/c mice injected with (**a**) [^99m^Tc]Tc-HYNIC-MGS5 (red) and (**b**) [^99m^Tc]Tc-HYNIC-MGS11 (blue) as analyzed 10 min p.i.; dashed line showing the radiochromatogram of the radiopeptide before injection.

Metabolic studies in BALB/c mice were performed to further evaluate the enzymatic stability of the radiopeptides. In these studies differences in the stability against enzymatic degradation could be observed between the two radiopeptides. For [^99m^Tc]Tc-HYNIC-MGS5 >65% intact radiopeptide was found in the blood and liver of BALB/c mice at 10 min after injection. Interestingly, the stability against metabolic degradation was further increased for [^99m^Tc]Tc-HYNIC-MGS11. The values of intact radiopeptide at 10 min p.i. were 96.0 ± 1.4% in blood and 95.2 ± 0.7% in liver. A faster degradation was observed in the excretory system. Much lower levels of intact radiopeptide were detectable in kidneys ([^99m^Tc]Tc-HYNIC-MGS5: 13.9 ± 0.5%; [^99m^Tc]Tc-HYNIC-MGS11: 40.0 ± 2.0%) and urine ([^99m^Tc]Tc-HYNIC-MGS5: 3.0 ± 1.6%; [^99m^Tc]Tc-HYNIC-MGS11: 9.8 ± 2.2%). Peptide related impurities already present after radiolabeling and with a relative retention of 0.9–1.1 were not considered as metabolites formed during digestion. Representative radio-HPLC profiles of the different analyzed samples are presented in Figure 6. For [^99m^Tc]Tc-HYNIC-MGS5 two main metabolites were observed in blood and liver, an early eluting hydrophilic metabolite with retention time (t_R_) of ~3.5 min and a second more hydrophobic metabolite eluting at t_R_ ~15.7 min. For [^99m^Tc]Tc-HYNIC-MGS11 only the metabolite with t_R_ ~3.5 min was detected whereas the second metabolite was missing. This indicates that additional methylation of the peptide bond between Asp and 1-Nal prevents the formation of this metabolite during systemic circulation. No further investigations have been performed yet to identify the metabolite found for [^99m^Tc]Tc-HYNIC-MGS5. From the shift in retention time between [^99m^Tc]Tc-HYNIC-MGS5 (t_R_ = ~17.5 min) and the formed metabolite (t_R_ = ~15.7 min) as well as from data available on the literature cleavage at two different positions, namely between (*N*-Me)Nle-Asp or Asp-1-Nal, seems possible. 

Ocak et al. have suggested a common enzymatic cleavage site of different MG analogs between Asp and Phe-NH2 when incubated in human serum [26]. Recently, Sauter et al. studied the stability of three ^177^Lu-labeled MG analogs in different human proteases finding that substitution of Met with Nle had a stabilizing effect and only cleavage between Nle and Asp occured [18]. In kidney and urine a much higher degree of enzymatic degradation was observed for [^99m^Tc]Tc-HYNIC-MGS5 and [^99m^Tc]Tc-HYNIC-MGS11. In kidneys mainly the hydrophilic metabolite with t_R_ ~3.5 min not corresponding to pertechnetate eluting at t_R_ 2.6 min was observed, whereas in urine a variety of additional metabolites with t_R_ < 15 min was detected.

### 2.5. Biodistribution in Tumor-xenografted BALB/c Nude Mice 

**Figure 7:**
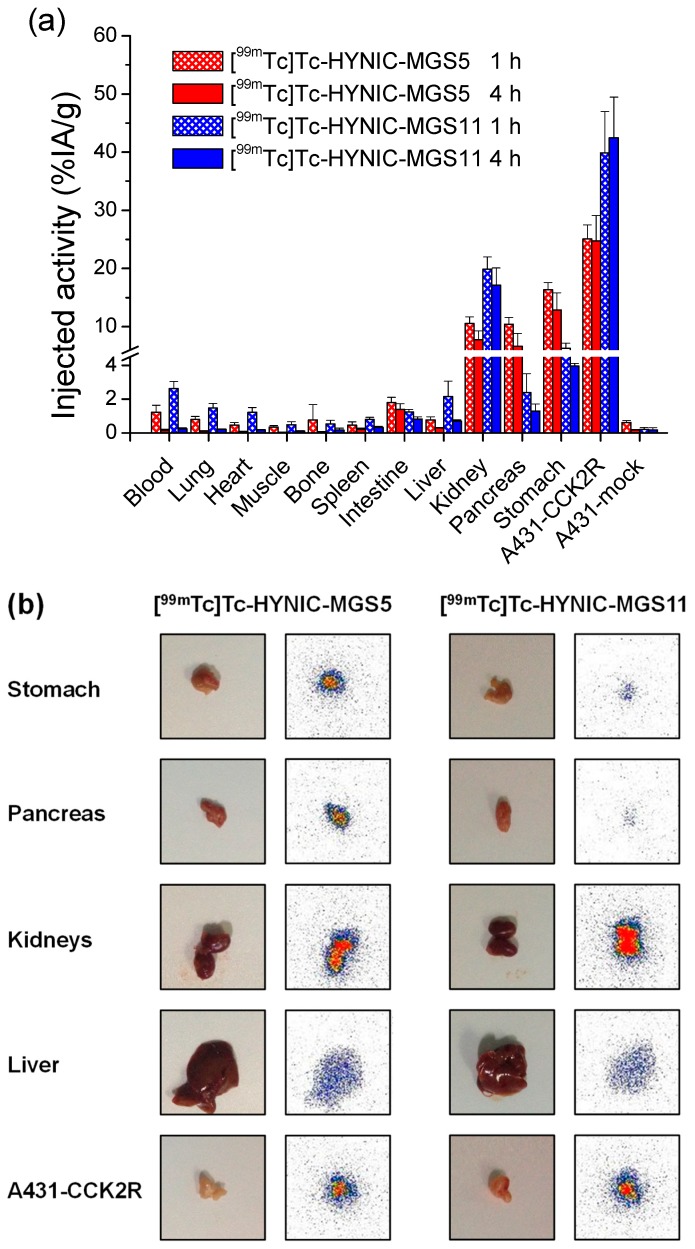
Biodistribution of [^99m^Tc]Tc-HYNIC-MGS5 (red) and [^99m^Tc]Tc-HYNIC-MGS11 (blue) in the A431-CCK2R/A431-mock xenograft model: (**a**) tissue distribution and tumor uptake at 1 h and 4 h p.i. with values expressed as % IA/g (mean ± SD, *n* = 4); (**b**) autoradiography performed for selected organs at 1 h p.i. (color scale, pixel intensity: min 9 (blue), max 100 (red)).

The results of the biodistribution studies in the A431-CCK2R/A431-mock tumor-xenograft model at 1 h and 4 h p.i. evaluating the tumor targeting and tissue uptake are displayed in Figure 7a. For selected organs additional autoradiography studies were performed at 1 h p.i. (see Figure 7b). The uptake values calculated for the tumors and different dissected tissues are summarized in the supplementary material (Table S1). Rapid clearance from the body mainly through the kidneys resulted in low non-specific uptake in most tissues and organs for [^99m^Tc]Tc-HYNIC-MGS5 as well as for [^99m^Tc]Tc-HYNIC-MGS11. The uptake of both radiopeptides significantly decreased in most tissues from 1 h to 4 h p.i. (*p* < 0.05) except for stomach (*p* = 0.07), intestine (*p* = 0.14) and bone (*p* = 0.18) in mice injected with [^99m^Tc]Tc-HYNIC-MGS5 as well as pancreas (*p* = 0.12) and kidneys (*p* = 0.18) in mice injected with [^99m^Tc]Tc-HYNIC-MGS11. [^99m^Tc]Tc-HYNIC-MGS5 showed a significantly lower uptake in blood at 1 h in comparison with [^99m^Tc]Tc-HYNIC-MGS11 (*p* < 0.01), however both radiopeptides showed a similar hydrophobicity and protein binding. At 4 h p.i. a similar trend was observed in blood, but this was not significant (*p* = 0.06). Also the non-specific uptake in lung, heart, spleen and liver was higher for [^99m^Tc]Tc-HYNIC-MGS11 at 1 h and 4 h p.i. (*p* < 0.05). In mouse CCK2R are primarily localized in brain and stomach and at lower expression levels also in colon, pancreas, kidney and ovary [31]. Due to the overall negative charge the two radioligands are unable to cross the blood-brain barrier, but we found a considerable uptake in stomach and pancreas, which was significantly higher for [^99m^Tc]Tc-HYNIC-MGS5 at both time points. For [^99m^Tc]Tc-HYNIC-MGS5 at 4 h p.i. a stomach uptake of 12.89 ± 2.91% IA/g and a pancreas uptake of 6.64 ± 2.21% IA/g was found, whereas [^99m^Tc]Tc-HYNIC-MGS11 displayed much lower values of 3.95 ± 0.15% IA/g in stomach (*p* = 0.0009) and 1.30 ± 0.42% IA/g in pancreas (*p* = 0.003) at the same time point. Also the intestinal uptake of [^99m^Tc]Tc-HYNIC-MGS11 with values of 0.82 ± 0.14% IA/g versus 1.39 ± 0.34% IA/g for [^99m^Tc]Tc-HYNIC-MGS5 at 4 h p.i. was significantly lower (*p* = 0.02). In line with the high CCK2R expression level confirmed for A431-CCK2R cells [32], an impressively high CCK2R mediated tumor uptake was observed in A431-CCK2R xenografts, whereas the uptake in A431-mock xenografts remained at very low levels (<1% IA/g). Tumor weights as determined after sacrifice were 0.17 ± 0.06 g for A431-CCK2R xenografts (*n* = 16) and 0.16 ± 0.12 g for A431-mock xenografts (*n* = 15). One mouse did not develop the A431-mock tumor. In A431-CCK2R xenografts, [^99m^Tc]Tc-HYNIC-MGS5 showed a high and persistent uptake with values of 25.09 ± 2.39% IA/g at 1 h and 24.75 ± 4.38% IA/g at 4 h p.i. The uptake values of [^99m^Tc]Tc-HYNIC-MGS11 were almost doubled (39.87 ± 7.12% IA/g at 1 h and 42.48 ± 6.99% IA/g at 4 h p.i.; *p* < 0.01). This improvement was rather surprising given the lower cell uptake observed in vitro (24.4 ± 1.9% for [^99m^Tc]Tc-HYNIC-MGS11 versus 62.0 ± 1.6% for [^99m^Tc]Tc-HYNIC-MGS5 after 2 h incubation). Receptor specificity was only tested by blocking studies *in vitro*, however, the very low uptake values observed in A431-mock tumor-xenografts (0.17–0.93% IA/g) for both radioligands confirmed that the tumor uptake was highly receptor specific. A very low and similar peptide amount of ~15 pmol was injected to the animals studied with both radiopeptides to avoid possible receptor saturating effects. The divergent uptake values observed for mouse stomach and pancreas in comparison with A431-CCK2R tumor-xenografts observed with both radioligands are therefore rather related to interspecies receptor differences between mouse and human CCK2R, than varying receptor expression levels. Even though the mouse and human CCK2R share an amino acid identity of ~90% [31], methylation of the peptide bond between Asp and 1-Nal in [^99m^Tc]Tc-HYNIC-MGS11 seems to affect the uptake into CCK2R-expressing organs in mouse. Interspecies differences between human and rat CCK2R have been reported also for other radiolabeled MG analogs [15,33]. The observed differences in stomach and pancreas need to be interpreted with caution as a different physiological uptake may occur in humans. However, no further experiments were carried out to investigate the affinity of the two MG analogs for mouse CCK2R.

The structural difference of the two radioligands also had a strong impact on the kidney uptake. The kidney uptake of [^99m^Tc]Tc-HYNIC-MGS11 (19.90 ± 2.09% IA/g at 1 h and 17.17 ± 2.93% IA/g at 4 h p.i.) was two times higher in comparison with [^99m^Tc]Tc-HYNIC-MGS5 (10.56 ± 1.15% IA/g at 1 h and 7.80 ± 1.47% IA/g at 4 h p.i.). Despite CCK2R-related uptake, reabsorption and retention of radiolabeled peptides in kidneys is mainly driven by multiple transport mechanisms, involving megalin and cubulin [34]. In megalin-deficient mice a significantly reduced renal reabsorption was confirmed for a radiolabeled minigastrin analog, with renal uptake values reduced to 37–49% when compared to wild-type mice [35]. It has been shown for different radiolabeled MG analogs that the uptake in stomach and CCK2R-expressing tumor-xenografts can be efficiently blocked by co-injection of a 1000-fold molar excess of unlabeled peptide, whereas no considerable effect occurs in kidneys [36]. In this study no additional blocking studies were performed in vivo to confirm the receptor-specific uptake in the different organs.

The tumor targeting profile of [^99m^Tc]Tc-HYNIC-MGS5 well compares with previous results obtained with [^111^In]In-DOTA-MGS5, showing a similar tumor uptake of 19.53 ± 5.42% IA/g and 23.49 ± 1.25% IA/g at 1 h and 4 h p.i., respectively [15]. This high and persistent tumor uptake is clearly superior when compared to other ^99m^Tc-labeled MG analogs previously studied [37]. For [^99m^Tc]Tc-Demogastrin-2 evaluated in A431-CCK2R xenografted SCID mice at a similar injected peptide amount of 10 pmol a tumor uptake of 12.89 ± 4.69% ID/g at 4 h p.i. was reported, which was however, connected with a very high kidney uptake (58.62 ± 8.98% ID/g). The metabolic stability of this compound (60% intact radiopeptide at 5 min p.i.) was comparable to [^99m^Tc]Tc-HYNIC-MGS5 [37]. In our previous studies with radiolabeled DOTA-MGS5 we concluded that a combination of increased protein binding and stabilization against enzymatic degradation might be responsible for the highly improved targeting profile. The enhanced protection against metabolic degradation of [^99m^Tc]Tc-HYNIC-MGS11 led to a further doubling in tumor uptake. Such an improvement could not be achieved by enzymatic stabilization alone, as exemplified by the co-injection of protease inhibitors [37]. [^99m^Tc]Tc-Demogastrin-2 coinjected with 300 µg phosphoramidon showed a similar enzymatic stability in vivo (85% intact radiopeptide at 5 min p.i.) as compared to [^99m^Tc]Tc-HYNIC-MGS11, but the tumor uptake was clearly inferior (18.21 ± 5.97% ID/g at 4 h p.i.). When comparing the tumor-to-organ activity ratios of [^99m^Tc]Tc-HYNIC-MGS5 and [^99m^Tc]Tc-HYNIC-MGS11, somewhat lower tumor-to-blood ratios were observed for [^99m^Tc]Tc-HYNIC-MGS11. Thus, [^99m^Tc]Tc-HYNIC-MGS11 also showed increased non-specific uptake in most organs at both investigated time points. Due to the concomitant increase of the uptake in A431-CCK2R xenografts and kidneys observed for [^99m^Tc]Tc-HYNIC-MGS11, a similar tumor-to-kidney ratio was found for both radiopeptides (2.4–3.3 for [^99m^Tc]Tc-HYNIC-MGS5 and 2.0–2.6 for [^99m^Tc]Tc-HYNIC-MGS11). The respective tumor-to-organ activity ratios calculated for blood, kidney, intestine, pancreas and stomach for both radioligands at different time points are displayed in Table 1.

To our knowledge this is the first report on ^99m^Tc-labeled CCK2R targeting peptide analogs showing such an astonishingly improved tumor uptake along with clearly reduced kidney uptake. [^99m^Tc]Tc-HYNIC-MGS5 showed a very similar targeting profile when compared to DOTA-MGS5 radiolabeled with different radiometals suitable for SPECT, PET and TRT [15]. The tumor uptake of [^99m^Tc]Tc-HYNIC-MGS11 was further improved, however, connected with a concomitant increase in kidney uptake. Recently, two ^99m^Tc-labeled non-peptidic radioligands have been described showing high tumor uptake in a mouse tumor model based on human epithelial cells transfected with CCK2R, however, tumor-to-kidney ratio was clearly inferior [38,39]. 

These developments give high promise that in the near future a kit for ^99m^Tc-labeling will be available allowing the localization and staging of CCK2R expressing tumors. Gastrin receptor scintigraphy might show a lower sensitivity when compared to PET imaging with a ^68^Ga-labeled CCK2R targeting MG analog, but additionally allows for radioguided surgery. The concept of surgical guidance with conventional gamma probes for intraoperative identification and removal of metastatic lesions has already been successfully introduced into clinical practice by the use of ^99m^Tc-labeled somatostatin analogs in patients with neuroendocrine tumors [40] as well as ^99m^Tc-labeled ligands targeting prostate specific membrane antigen in patients with prostate cancer [41]. Due to its high tumor uptake and tumor retention combined with high clearance from other tissues [^99m^Tc]Tc-HYNIC-MGS11 might be favorable for imaging and radioguided surgery. [^99m^Tc]Tc-HYNIC-MGS5 well compares with DOTA-MGS5 and shows the advantage of using the same peptide for different applications, [^68^Ga]Ga-DOTA-MGS5 for PET, [^99m^Tc]Tc-HYNIC-MGS5 for SPECT and radioguided surgery, as well as [^177^Lu]Lu-DOTA-MGS5 for TRT.

## 3. Materials and Methods

### 3.1. Materials

All commercially obtained chemicals were of analytical grade and used without further purification. Na[^99m^Tc]TcO_4_ was obtained from a commercial ^99^Mo/^99m^Tc-generator (Ultratechnekow, Mallinckrodt, Petten, The Netherlands) eluted with physiological saline. The A431 human epidermoid carcinoma cell line stably transfected with the plasmid pCR3.1 containing the full coding sequence for the human CCK2R (A431-CCK2R) as well as the same cell line transfected with the empty vector alone (A431-mock) were kindly provided by Dr. Luigi Aloj [42]. Both cell lines were cultured in Dulbecco’s Modified Eagle Medium (DMEM) supplemented with 10% (*v*/*v*) fetal bovine serum and 5 mL of a 100× penicillin-streptomycin-glutamine mixture at 37 °C in a humidified 95% air/5% CO_2_ atmosphere. Media and supplements were purchased from Invitrogen Corporation (Lofer, Austria).

### 3.2. Peptide Synthesis

HYNIC-dGlu-Ala-Tyr-Gly-Trp-(*N*-Me)Nle-Asp-1-Nal-NH_2_ (HYNIC-MGS5) and HYNIC-dGlu-Ala-Tyr-Gly-Trp-(*N*-Me)Nle-Asp-(*N*-Me)1-Nal-NH_2_ (HYNIC-MGS11) were synthesized using 9-fluorenylmethoxycarbonyl (Fmoc) chemistry. The peptides were assembled on 60 mg Rink Amide MBHA resin with capacity 0.5 mmol/g resin (Novabiochem, Hohenbrunn, Germany). The reactive side chains of the amino acids were masked with the following protection groups: tert-butyl ester for Asp and dGlu, tert-butyl ether for Tyr, and tertbutyloxycarbonyl (BOC) for Trp. All coupling reactions were performed using a 5-fold excess of Fmoc-protected amino acids, 1-hydroxy-7-aza-benzotriazole (HOAt) and *O*-(7-Azabenzotriazole-1-yl)-*N*, *N*,*N*’*N*’-tetramethyluronium hexa-fluorophosphate (HATU) in *N*-Methyl-2-pyrrolidone (NMP) pH adjusted to 8-9 with *N*,*N*’-diisopropylethylamine. Coupling of the Fmoc-protected amino acids following (*N*-Me)Nle or (*N*-Me)1-Nal was repeated twice. For the coupling of HYNIC a 3-fold molar excess of BOC-HYNIC, HOAt and HATU was used. The introduction of an additional methyl group into the peptide bound between Asp and 1-Nal in HYNIC-MGS11 was performed by direct *N*-methylation of 1-Nal during the peptide synthesis on the solid resin as described by Chatterjee et al. [43]. Cleavage of the peptides from the resin with concomitant removal of acid-labile protecting groups was achieved by treatment with a mixture of trifluoroacetic acid (TFA), triisopropylsilane, and water in a ratio 95/2.5/2.5 *v*/*v*/*v*. The crude peptides were precipitated and washed with ether before HPLC purification and characterized by analytical HPLC and MALDI-TOF MS. The lyophilized peptide derivatives were stored at −20 °C.

### 3.3. Analytical Systems and Methods

For preparative HPLC purification a Gilson 322 chromatography system (Gilson International, Limburg, Germany) with Gilson UV/VIS-155 multi-wavelength detector, equipped with an Eurosil Bioselect Vertex Plus C18A precolumn (300 Å, 5 μm, 30 × 8 mm) and a Eurosil Bioselect Vertex Plus C18A column (300 Å 5 μm 300 × 8 mm) (Knauer, Berlin, Germany) was used with a gradient system starting from 80% solvent A (water containing 0.1% TFA) and increasing concentrations of solvent B (acetonitrile (ACN) containing 0.1% TFA) with a flow rate 2 mL/min: 0–4 min 20% B, 4–24 min 20–60% B, 24–26 min 60% B, 26–27 min 60–80% B, 27–28 min 80% B, 28–29 min 80–20% B, 29–37 min 20% B.

Analytical HPLC was performed using an UltiMate 3000 chromatography system (Dionex, Germering, Germany) consisting of a HPLC pump, a variable UV-detector (UV-VIS at λ = 280 nm), an autosampler, a radiodetector (GabiStar, Raytest, Straubenhardt, Germany), equipped with a Phenomenex Jupiter 4 µm Proteo C12 90 Å 250 × 4.6 mm column (Phenomenex Ltd., Aschaffenburg, Germany) using a flow rate of 1 mL/min together with the following gradient system: 0–3 min 10% B, 3–18 min 10–55% B, 18–20 min 80% B, 20–21 min 80–10% B, 21–25 min 10% B.

For Matrix Assisted Laser Desorption Ionization Time-of-Flight Mass Spectrometry (MALDI-TOF MS) a Bruker microflex benchtop MALDI-TOF MS (Bruker Daltonics, Bremen, Germany) was used in reflector acquisition mode with a positive ion source and 200 shots per spot. MALDI samples were prepared on a α-cyano-4-hydroxycinnamic acid (HCCA) matrix using dried droplet procedure. Flex Analysis 2.4 software was used to analyze the recorded data.

### 3.4. ^99m^Tc-Radiolabeling Using the Tricine/EDDA Exchange Method

^99m^Tc-labeling was performed using a previously described exchange labeling approach with tricine and EDDA [27]. For this purpose 10–20 µg of the corresponding HYNIC-conjugated peptide analog (dissolved in EtOH/H_2_O 30/70 *v*/*v* at a concentration of 0.5 µg/µL) together with 250 µL of EDDA solution (20 mg/mL in 0.1 M NaOH), 250 µL tricine solution (40 mg/mL in 0.2 M PBS pH 6), 500 µL of Na[^99m^Tc]TcO_4_ (≤750 MBq) and 20 μl of tin(II) chloride solution (20 mg of SnCl_2_ * 2 H_2_O in 10 mL 0.1 N HCl), were incubated in a sealed glass vial at 100 °C for 15–20 min. Radiochemical purity of [^99m^Tc]Tc-HYNIC-MGS5 and [^99m^Tc]Tc-HYNIC-MGS11 was determined by analytical HPLC. For in vivo assays the radiolabeled peptides were purified by SPE. For this purpose, the labeling mixture was passed through a C18-SepPak-Light cartridge (Waters, Milford, MA, USA), followed by 5 mL saline, and the radiolabeled peptide was eluted with EtOH/H_2_O 65/35 *v*/*v* and diluted with PBS.

### 3.5. Evaluation of the in Vitro Properties

The resistance against degradation of [^99m^Tc]Tc-HYNIC-MGS5 and [^99m^Tc]Tc-HYNIC-MGS11 (1000 pmol/mL, *n* = 2) in human serum was studied for up to 24 h. Furthermore, the radiopeptides were incubated in PBS (1000 pmol/mL, *n* = 1). At each time point of 1, 2, 4 and 24 h after incubation the intact radiopeptide was assessed by analytical HPLC. Serum samples were precipitated with ACN and centrifuged to collect the supernatant and diluted with water prior to radio-HPLC. For the determination of the distribution coefficient (log D), 500 µL of the radiopeptide solutions (50 pmol/mL in PBS) were added to 500 µL octanol (1:1) vigorously vortexed (*n* = 8) for 15 min and centrifuged to separate the two phases. From each phase a 75 µL sample was taken, the radioactivity measured in a 2480 Wizard^2^ automatic gamma-counter (PerkinElmer Life Sciences and Analytical Sciences, Wallac Oy, Turku, Finland) and the distribution of the radiopeptides calculated. The protein binding in human serum (500 pmol/mL, *n* = 2) was assessed by Sephadex G-50 size-exclusion chromatography (GE Healthcare Illustra, Little Chalfont, UK) for up to 24 h.

### 3.6. Receptor Binding and Cell Internalization Studies

The receptor affinity of the radioligands prepared using the above described labeling protocol was evaluated in saturation studies on A431-CCK2R cells. For the assay, 96-well filter plates (MultiScreen_HTS_-FB, Merck Group, Darmstadt, Germany) were pretreated with 10 mM TRIS/139 mM NaCl buffer, pH 7.4 (TRIS-buffer) (2 × 250 μL) and 400,000 A431-CCK2R cells per well were added in 35 mM HEPES buffer, pH 7.4, containing 10 mM MgCl_2_, 14 μM bacitracin, and 0.5% bovine serum albumin (BSA), a hypotonic solution disturbing the integrity of the cell membranes. Thereafter, increasing concentrations of the radiolabeled peptide conjugates (0.1–112 nM) were added in triplicate reaching a total volume of 200 µL. In parallel, non-specific binding was determined by co-incubation with 1 µM pentagastrin. After 1 h incubation at room temperature, the medium was removed by filtration followed by two rapid rinses with ice-cold TRIS buffer (200 μL). The filters were collected and counted in a gamma-counter. The *K*_d_ value was calculated fitting the data with Origin software (Origin 6.1, OriginLab Corporation, Northampton, MA, USA) to a one-site binding model using the formula y = B_max_ × x/(*K*_d_ + x).

For internalization experiments, A431-CCK2R and A431-mock cells were seeded at a density of 1.0 × 10^6^ cells per well in 6-well plates and grown to confluence for 48 h. At the day of the experiment, cells were washed twice with ice-cold internalization medium supplemented with 1% (*v*/*v*) fetal bovine serum and supplied with fresh medium before incubation with [^99m^Tc]Tc-HYNIC-MGS5 and [^99m^Tc]Tc-HYNIC-MGS11 at a final peptide concentration of 0.4 nM in a total volume of 1.5 mL in triplicates. At different time points for up to 2 h incubation the cell uptake was interrupted by removal of the medium and rapid rinsing with ice-cold internalization medium (two times). Thereafter, the cells were incubated twice at ambient temperature in acid wash buffer (50 mM glycine buffer pH 2.8, 0.1 M NaCl) for 5 min, to remove the membrane-bound radioligand. Finally, the cells were lyzed by treatment in 1 M NaOH and collected (internalized radioligand fraction). All collected fractions (supernatant, surface wash, lyzed cells) were measured together with a standard in the gamma counter. The radioactivity of the lyzed cells was expressed as percentage of the total radioactivity added (% of internalized radioactivity). Non-specific binding was evaluated in A431-mock cells and in additional blocking studies with 1 µM pentagastrin.

### 3.7. Evaluation of the in Vivo Stability and Biodistribution

All animal experiments were conducted in compliance with the Austrian animal protection laws and with the approval of the Austrian Ministry of Science (BMWFW-66.011/0075-WF/V/3b/2016).

#### 3.7.1. Metabolic Stability in BALB/c Mice

Metabolic stability studies in vivo with [^99m^Tc]Tc-HYNIC-MGS5 and [^99m^Tc]Tc-HYNIC-MGS11 were performed in 5–6 week-old female BALB/c mice (Charles River, Sulzfeld, Germany; *n* = 2). Mice were injected intravenously via a lateral tail vain with 37–74 MBq of the ^99m^Tc-labeled HYNIC-analogs (corresponding to 2 nmol total peptide). Ten minutes post injection (p.i.) mice were euthanized and a sample of blood and urine was collected together with the liver and kidneys. Liver and kidneys were rapidly homogenized in 0.5 mL of a 20 mM HEPES buffer pH 7.3 with an Ultra-Turrax T8 homogenator (IKA-Werke, Staufen, Germany) for 1 min at RT. Before determining the percentage of intact radiopeptide by analytical radio-HPLC samples were precipitated with ACN, centrifuged and diluted with H_2_O (1:1).

#### 3.7.2. Biodistribution in Tumor-xenografted BALB/c Nude Mice

Biodistribution studies evaluating the tumor uptake of [^99m^Tc]Tc-HYNIC-MGS5 and [^99m^Tc]Tc-HYNIC-MGS11 were performed in 7 week-old female athymic BALB/c nude mice (Charles River, Sulzfeld, Germany). To induce tumor-xenografts, mice were injected subcutaneously with 2 × 10^6^ A431-CCK2R (right flank) and A431-mock cells (left flank). The tumor-xenografts were allowed to grow for 11–12 days reaching medium tumor weights of ~0.2 g. Mice were randomly divided into groups of four and injected intravenously via a lateral tail vein with 0.3 MBq of the ^99m^Tc-labeled HYNIC-analogs (corresponding to ~15 pmol total peptide). The groups of animals were sacrificed at 1 h and 4 h p.i., tumors and other tissues (blood, lung, heart, muscle, bone, spleen, intestine, liver, kidney, stomach and pancreas) were removed, weighed, and the radioactivity measured together with a standard in the gamma counter. Results were expressed as percentage of injected activity per gram tissue (% IA/g) and tumor-to-organ activity ratios were calculated for selected tissues. Statistical analysis was performed using independent two population t-test (significance level *p* = 0.05) with Origin software. The radioactivity in selected organs (stomach, pancreas, liver, kidneys, A431-CCK2R and A431-mock xenograft) was additionally visualized by autoradiography using a phosphorimager (Cyclone Plus, PerkinElmer Life Sciences and Analytical Sciences, Downers Grove, Il, USA). After dissection, the organs were exposed to a multisensitive storage phosphor screen for 40 min and image analysis was performed using OptiQuant^TM^ software (OptiQuant 5.0, PerkinElmer Life Sciences and Analytical Sciences, Downers Grove, Il, USA).

## Figures and Tables

**Table 1:** Tumor-to-organ activity ratios for A431-CCK2R tumor-xenografts of [^99m^Tc]Tc-HYNIC-MGS5 and [^99m^Tc]Tc-HYNIC-MGS11 (mean ± SD, *n* = 4).

	[^99m^Tc]Tc-HYNIC-MGS5		[^99m^Tc]Tc-HYNIC-MGS11	
	1 h p.i.	4 h p.i.	1 h p.i.	4 h p.i.
Tumor/blood	21.6 ± 5.1	273 ± 151	15.6 ± 4.5	177 ± 55
Tumor/kidney	2.4 ± 0.3	3.3 ± 1.1	2.0 ± 0.6	2.6 ± 0.8
Tumor/stomach	1.5 ± 0.2	2.0 ± 0.4	6.5 ± 1.9	10.7 ± 1.4
Tumor/pancreas	2.4 ± 0.3	4.2 ± 2.0	20.6 ± 11.7	34.8 ± 9.7
Tumor/intestine	14.2 ± 1.7	19.2 ± 7.5	32.3 ± 5.0	61.1 ± 14.3

## Supplementary Materials

The following are available online at http://www.mdpi.com/1424-8247/12/1/13/s1, Table S1: Biodistribution of [^99m^Tc]Tc-HYNIC-MGS5 and [^99m^Tc]Tc-HYNIC-MGS11 in BALB/c nude mice tumor-xenografted with A431-CCK2R and A431-mock tumors (mean ± SD, *n* = 4).
