# Correction: Klingler, M., et al. Cholecystokinin-2 Receptor Targeting with Novel C-terminally Stabilized HYNIC-Minigastrin Analogs Radiolabeled with Technetium-99m. *Pharmaceuticals* 2019, *12*, 13

**DOI:** 10.3390/ph12020088

**Published:** 2019-06-13

**Authors:** Maximilian Klingler, Christine Rangger, Dominik Summer, Piriya Kaeopookum, Clemens Decristoforo, Elisabeth von Guggenberg

**Affiliations:** Department of Nuclear Medicine, Medical University of Innsbruck, Anichstrasse 35, A-6020 Innsbruck, Austria; maximilian.klingler@i-med.ac.at (M.K.); christine.rangger@i-med.ac.at (C.R.); summer.dominik@gmail.com (D.S.); piriya.kaeopookum@student.i-med.ac.at (P.K.); clemens.decristoforo@i-med.ac.at (C.D.)

In our paper [1], an error unfortunately occurred in Figure 1. We therefore would like to provide the readers with the corrected figure and apologize for this inconvenience.

## Figures and Tables

**Figure 1:**
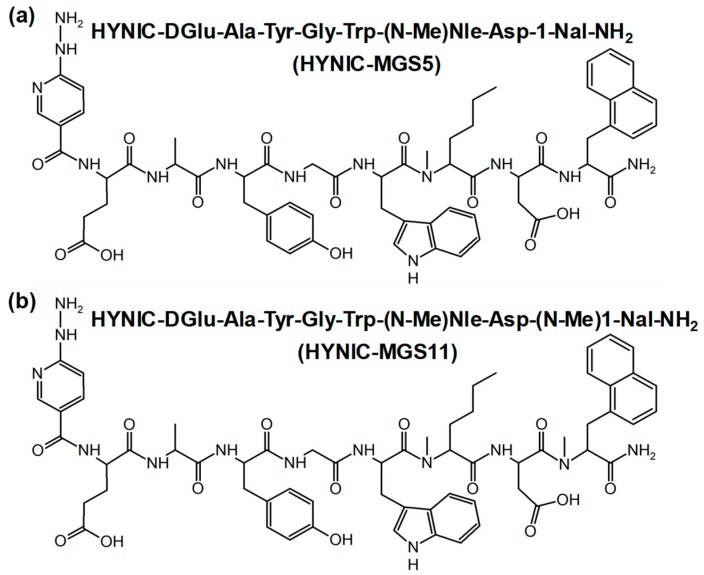
Amino acid sequence and chemical structure of (**a**) HYNIC-MGS5 and (**b**) HYNIC-MGS11.
