# Supplementary material for: Cholecystokinin-2 Receptor Targeting with Novel C-terminally Stabilized HYNIC-Minigastrin Analogs Radiolabeled with Technetium-99m
Source: Pharmaceuticals (Basel). 2019 Jan 15;12(1):13. doi: 10.3390/ph12010013 (PMC6469167; doi:10.3390/ph12010013)
Supplement: Supplementary file 1 [file pharmaceuticals-12-00013-s001.pdf]

pharmaceuticals

Table S1. Biodistribution of [^{99m}Tc]Tc-HYNIC-MGS5 and [^{99m}Tc]Tc-HYNIC-MGS11 in BALB/c nude mice tumor-xenografted with A431-CCK2R and A431-mock tumors (mean±SD, n=4).

	**[^{99m}Tc]Tc-HYNIC-MGS5**		**[^{99m}Tc]Tc-HYNIC-MGS11**	
	**1 h p.i.**	**4 h p.i.**	**1 h p.i.**	**4 h p.i.**
Blood	1.23±0.41	0.13±0.09	2.62±0.44	0.25±0.05
Lung	0.79±0.19	0.11±0.02	1.47±0.27	0.20±0.05
Heart	0.47±0.14	0.07±0.02	1.22±0.28	0.18±0.03
Muscle	0.34±0.10	0.04±0.02	0.48±0.19	0.09±0.05
Bone	0.77±0.91	0.07±0.01	0.53±0.22	0.17±0.12
Spleen	0.47±0.19	0.21±0.08	0.81±0.12	0.33±0.06
Intestine	7.79±0.32	1.39±0.34	1.24±0.15	0.82±0.14
Liver	0.76±0.20	0.28±0.05	2.15±0.92	0.70±0.08
Kidney	10.56±1.15	7.80±1.47	19.90±2.09	17.17±2.93
Pancreas	10.44±1.16	6.64±2.21	2.39±1.12	1.30±0.42
Stomach	16.34±1.26	12.89±2.91	6.29±0.89	3.95±0.15
Tumor xenograft				
A431-CCK2R	25.09±2.39	24.75±4.38	39.87±7.12	42.48±6.99
A431-mock	0.62±0.11 [1]	0.17±0.03	0.20±0.13	0.16±0.16

[1] In this group one mouse did not develop the A431-mock xenograft.
